# A High-Energy Diet and Spirulina Supplementation during Pre-Gestation, Gestation, and Lactation do Not Affect the Reproductive and Lactational Performance of Primiparous Sows

**DOI:** 10.3390/ani12091171

**Published:** 2022-05-03

**Authors:** Rosamaria Lugarà, Łukasz Grześkowiak, Jürgen Zentek, Susanne Meese, Michael Kreuzer, Katrin Giller

**Affiliations:** 1 Animal Nutrition, ETH Zurich, Eschikon 27, 8315 Lindau, Switzerland; rosamaria.lugara@usys.ethz.ch (R.L.); michael.kreuzer@usys.ethz.ch (M.K.); 2 Institute of Animal Nutrition, Freie Universität Berlin, Königin-Luise-Strasse 49, 14195 Berlin, Germany; lukasz.grzeskowiak@fu-berlin.de (Ł.G.); juergen.zentek@fu-berlin.de (J.Z.); 3 Vetsuisse Faculty, Clinic of Reproductive Medicine, University of Zurich, Eschikon 27, 8315 Lindau, Switzerland; susanne.meese@uzh.ch

**Keywords:** primiparous sow, dietary fat, microalga, offspring sex ratio, colostrum composition, pre-weaning mortality

## Abstract

**Simple Summary:**

High-energy or high-fat diets are often fed to pregnant and lactating sows to overcome problems resulting from insufficient energy intake. However, their long-term consumption has the potential to impair the reproductive and lactational traits in sows. The microalga spirulina has been previously described to exert beneficial health effects, and it may potentially reverse the detrimental effects induced by a high-energy diet. In the present study, both a high-energy diet and the spirulina did not affect the reproduction and lactational traits in sows, though they both influenced the colostrum fatty acids profile in a way that may impact offspring growth and health. Moreover, sows fed a high-energy diet tended to have less piglets weaned than sows fed a control diet. The spirulina was not able to reverse these effects but tended to affect the proportion of males and females per litter in a diet-specific manner.

**Abstract:**

Feeding high-energy (HED) or high-fat diets during gestation and lactation to pigs may help cover the energy requirements of high-prolific sows but may also adversely affect their reproductive performance. The microalga *Arthrospira plantensis* (Sp), rich in bioactive compounds, has been described to exert beneficial health effects. The present study investigated the effects of HED and Sp intake during gestation and lactation in pigs. Twenty-four primiparous crossbred sows were fed either a HED or a control diet. Half of the sows per group were supplemented with 20 g/day of Sp. Despite a higher gross energy intake, consuming the HED did not affect the sows’ reproductive and lactational performance but significantly modified the colostrum fatty acid (FA) composition and tended to decrease the number of weaned piglets. The Sp supplementation did not affect the reproduction and lactation traits, but slightly affected the colostrum FA composition. A trend was observed for an interaction of diet and Sp in terms of offspring sex ratio with a 50% lower male-to-female ratio in the HED group compared to all other animals. These findings suggest that an HED and Sp intake hardly influence reproduction in sows. However, the HED modified the colostrum FA composition, whereas the Sp had only fewer effects, which may potentially affect offspring performance.

## 1. Introduction

Covering the nutrient requirements during pregnancy and lactation is crucial to ensure the optimal performance of sows in the long term. The selection of sows with a high prolificacy has resulted in bigger litters but also decreased piglet vitality and growth [1]. However, the spontaneous intake capacity may not follow the increased requirements for energy and nutrients, even under ad libitum access to the diet [2]. Accordingly, sows with a low energy intake during gestation show reduced pregnancy rates and embryonic survivals [3]. A strategy suggested to overcome such adverse effects is to increase the energy density of the diets for sows during gestation. Ensuring the high energy intake of sows, especially during both late gestation and lactation, may also prevent excessive body weight (BW) loss during lactation [3]. However, the balance in energy density is delicate because an excessive energy intake during the entire gestation may lead to an increased BW that often results in complications at farrowing [4]. In addition, overly heavy sows show decreased feed intake during early lactation and excessive BW loss with detrimental effects on reproductive performance, especially longer weaning-to-estrus intervals, lower pregnancy rates, increased embryo mortality, and decreased milk production [5,6,7,8]. The chronic consumption of high-energy diets may favor a pro-inflammatory state and increase oxidative stress in the organism, thereby impairing reproductive performance and milk production [9].

In comparison to the feed ingredients used in conventional sow diets, which are rich in polysaccharides such as starch and low in fiber, the energy density of the diet may be substantially enhanced by adding particularly energy-dense nutrients such as fat and sugars. The addition of dietary fat was shown to counterbalance the negative energy balance of early lactating sows and increase milk yield [10]. However, adding dietary fat can also lead to changes in colostrum and milk composition, which consequently modifies the diet of the suckling offspring and thus may affect their growth performance and health status, ultimately impairing their productivity and fertility [11]. Other than the effects of fat addition to the diet of sows, there is only scarce information about the addition of sugars and cholesterol.

Nutritional supplements may provide a useful tool to counteract any adverse effects of diets too dense or too low in energy on the performance of the sow and her litter. In other livestock species, a supplementation of the microalga *Arthrospira platensis* (also known as spirulina, Sp) indeed enhanced the reproductive performance of the animals [12,13] by improving the oxidative status of the animals, including an increased plasma total antioxidant capacity and an increase in the contents of glutathione S-transferase, superoxide dismutase, and glutathione peroxidase. Sp is rich in bioactive phytochemicals [14]. Among these, the pigments, such as carotenoids, tocopherols, and phycocyanin, are presumed to contribute to the antioxidant defense usually described for Sp (as reviewed by Wu et al. [15]). Spirulina also contains high proportions of bioactive long-chain *n*-6 fatty acids (FA), especially γ-linolenic acid (GLA) and linoleic acid (LA). The intake of such FAs during gestation and lactation may be beneficial for both mothers and the developing offspring [16]. The characteristic FA profile of Sp may also be reflected in the milk composition and thus affect the piglets’ postnatal development and health. So far, no study has investigated if Sp fed to gestating and lactating pigs may counteract or, at least, attenuate any adverse effects of a diet with too high or too low of an energy density.

Therefore, in the present study, we aimed to investigate how a high-energy diet (HED) in comparison to a commercial pig diet affects the reproductive and lactation performance of pigs and to test if low-dose Sp supplementation may attenuate such potential effects. We hypothesized that (i) feeding a diet particularly rich in fat and sugars to domestic pigs during gestation and lactation impairs reproductive and lactational performance; (ii) offering such a diet affects colostrum composition; and that (iii) dietary Sp supplementation counteracts any adverse effects of such a diet on reproductive and lactational performance.

## 2. Materials and Methods

### 2.1. Animals, Housing, and Experimental Protocol

**Figure 1:**
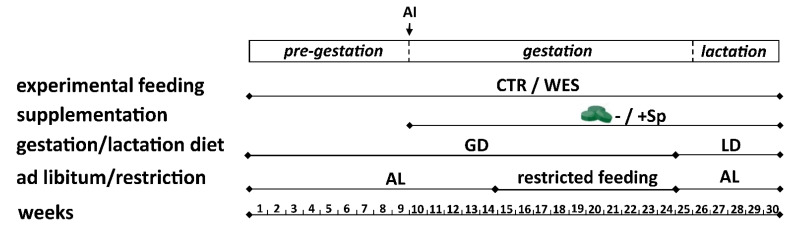
Schematic representation of the feeding experiment. AI: artificial insemination; AL: ad libitum; CTR: control diet; GD: gestation diet; LD: lactation diet; Sp: spirulina; WES: Western diet.

The experiment was performed at AgroVet-Strickhof (Lindau, Switzerland) in accordance with the Swiss legislation on animal rights and welfare with the approval of the Cantonal Veterinary Office of Zurich, Switzerland (license number ZH157/18). Because of a limited housing capacity, the experiment was performed in two identical runs, each carried out from spring to winter in 2019 and 2020, respectively. The ambient temperature was kept between 17 and 22 °C and the relative humidity was kept at 60%. Twenty-four nulliparous Large White × Swiss Landrace sows (*n* = 12 per run, all from the same sire) with an initial BW of 119 ± 8 kg (mean ± standard deviation (SD)) at an average age of 5.6 ± 0.8 months were obtained from a commercial pig farm. The sows were kept in pens in pairs. The pens were equipped with automatic feeding stations (Pig Performance Tester, Nedap, Groenlo, The Netherlands), which allowed time-independent access to feed and recorded the individual daily feed intake using ear-tag transponders. The sows had ad libitum access to water, to a container with compressed straw, and to straw used as housing-enrichment material. Details about the experimental design can be found in Figure 1. After 6–8 weeks of pre-gestational feeding with the respective experimental diets, estrus synchronization via an oral application of Regumate^®^ (MSD, New Animal Health, Wellington, New Zealand) was initiated. All sows in both runs were artificially inseminated (AI) with fresh sperm from one boar (PREMO^®^ Large White, Suisag, Sempach, Switzerland). After AI, feed was provided at ad libitum access until day 40, then was reduced to 2 and 3 kg/day from day 40 to day 90 and from day 91 to day 105 of gestation, respectively. This measure was intended to prevent problems at parturition due to excessive BW. On day 18 post AI, the sows were controlled for pregnancy via heat detection and on day 25 by ultrasonography (PigLOG 105, Frontmatec Kolding, Kolding, Denmark). Only sows found pregnant on day 25 were further included in the experiment. From day 44 of gestation onwards, sows were moved to individual farrowing pens (335 cm × 230 cm, ATX Suisse GmbH, Ermensee, Switzerland). The neighboring pens had a communicating door; thus, sows could be kept in pairs until farrowing. One week before the expected parturition (day 107 of gestation), the sows were separated and kept in farrowing pens until the offspring was weaned at about 4 weeks of age. These pens were equipped with a compartment accessible only to the piglets with a heated lid (35 °C), one feed and one water trough for the dams, and two water nipples for the piglets. From this time onwards, the diets of both groups were switched to a lactation type. This diet type was offered ad libitum to the sows until weaning.

### 2.2. Experimental Diets

All diets used in this experiment were produced by Weinlandmuehle Truellikon (Glanzmann AG, Truellikon, Switzerland). At the start of the pre-gestation period, the sows were randomly assigned to two groups (*n* = 12 per group) with a similar average BW, which received diets of different energy densities. These were either control gestation or lactation diet types (CTR group) or high-energy gestation or lactation diet types (HED group) (Table 1). The CTR types resembled standard commercial diets, designed to cover the basic nutritional requirements for gestating and lactating pigs (Table 1). The HED types were designed with a substantially increased energy density (+3.8 MJ gross energy/kg dry matter for both the gestation and lactation diets) realized by replacing wheat and part of the corn germ with hydrogenated palm oil (150 g/kg diet) and sugars (saccharose and D-fructose; 350 g/kg diet). For another experimental purpose (evaluating the effects of a Western diet on human metabolism with a pig as a model), a substantial amount of cholesterol was also added to the HED (Table 1). Three days before AI, the manual supplementation of 20 g/day of Sp in the form of tablets (IGV Planttech GmbH, Nuthetal, Germany) was started for half of the sows in the CTR and HED groups (-: without Sp; +: with Sp). This ultimately resulted in four experimental groups, namely CTR-, CTR+, HED-, and HED+. Supplementation was performed each morning by providing Sp on disposable plates placed on the floor to secure the individual intake. The Sp supplementation was continued until the end of the experiment.

### 2.3. Reproductive and Lactational Performance

Throughout gestation and lactation, individual feed and energy intake was determined on 2 days per week, either from data registered by the feeding stations in the gestation pens or by recording the amounts of supply and leftovers in the farrowing pens. For the calculation of the gestation length, farrowing was not artificially induced. At farrowing, the gestation length, litter size, and individual piglet birth weight and sex were recorded. In total, four piglets were excluded from the calculation of the average birth weight because of congenital disorders, showing only partial development. Standard litter traits, particularly the number of piglets born, piglets born alive, and fetal deaths were recorded at farrowing. Throughout the lactation period, the weekly BW and mortality of the piglets were recorded, and finally the number of piglets weaned was determined. The performance of the weaned piglets and their carcass and meat quality after completing the fattening period is described elsewhere [17].

The total milk production was estimated with the help of the BW development of all the piglets per litter during the suckling period by applying the equation presented by Miao et al. [18], reading:

Total milk yield (kg) = average daily gains of individual piglet × litter size × days of lactation × 4.

From these data, the average daily milk amount provided by the sows per piglet was calculated.

### 2.4. Composition of the Colostrum

At farrowing, after the birth of the first three to five piglets, colostrum samples (max. 30 mL) were collected by manual milking and were stored at −20 °C until further analyses. Crude fat and lactose were determined using the standard procedures of the Verband Deutscher Landwirtschaftlicher Untersuchungs- und Forschungsanstalten (VDLUFA) (VDLUFA VI 15.2.1, 20.2.3; [19]). The crude protein was analysed using the Dumas nitrogen determination method [20]. The concentrations of the immunoglobulins IgA and IgM in the colostrum were analysed using swine IgA and IgM ELISA quantification kits (Abcam, Cambridge, UK). The IgG was determined using a Pig IgG ELISA kit (Cat. No. E100-104, Bethyl Laboratories, Inc., Montgomery, TX, USA) with small modifications. Briefly, 100 µL of the colostrum was applied onto microtiter plates coated with goat anti-pig IgG antibodies (Bethyl Laboratories, Cat. No. A100-104A) and was incubated. After washing and subsequent treatments with goat anti-pig IgG-HRP antibodies (Bethyl Laboratories, A100-104P), TMB (Sigma-Aldrich, St. Louis, MO, USA, T0440), and 2M H2SO4, the optical density was measured at 450 nm. The IgG concentration in the samples was calculated based on a standard curve constructed from the purified pig IgG (Bethyl Laboratories, Cat. No. P100-105).

The gross energy content of the colostrum was calculated according to the formula used by Costermans et al. [8], reading:

Energy (kJ/kg) = (379.07 × fat (%) + 231.7936 × protein (%) + 972.78).

The FA analysis of the colostrum lipids was performed as described in detail by Ineichen et al. [21] for cow milk lipids. Briefly, internal standards (*n*-heptane containing triundecanoin, tetradecenoic methylate, and trivaleranoin) were mixed with the colostrum. Sodium methylate was used for cold transesterification to FAME [22]. Triglyceride standards (6:0, 13:0, and 19:0) were used to derive the response factors. A gas chromatograph (HP 6890, Agilent Technologies) equipped with a CP7421 column (200 m × 0.25 mm, Varian, Lake Forest, IL, USA) was used to separate the FAME. Peak identification was further confirmed using chromatograms from Collomb and Buehler [23].

### 2.5. Group sizes and Statistical Analyses

Group sizes differed between the different experimental periods. Originally, *n* = 12 sows per energy-density group were available at AI. At day 25 post AI, three non-pregnant sows were excluded, and one sow (HED+ group) had to be excluded because of an abortion on day 43 post AI. This eventually resulted in *n* = 5 for CTR-, *n* = 6 for CTR+, *n* = 5 for HED-, and *n* = 4 for HED+ for the gestation period. After farrowing, one CTR- sow was excluded from analyses due to agalactia in the lactation period (thus, *n* = 4 for CTR- in the lactation period).

All statistical analyses were performed using R (version 4.1.2) [24]. Linear mixed models were created using the packages lmerTest and lme4 (version 3.1-0 and 1.1-23, respectively). Diet, spirulina supplementation, and their nested interaction were considered as fixed effects. Sow, the year of the experiment, and the sow’s dam were included as random effects. For the analysis of the number of weaned piglets, the respective litter size was included as a covariate. A normal distribution was confirmed by graphically inspecting the residuals and by using the Shapiro–Wilk test. When the fixed effects met statistical significance, the normalized datasets were submitted to pairwise comparisons among least squares means using the emmeans package (v1.6.0). Differences were considered significant at *p* ≤ 0.050 and a trend at 0.051 ≤ *p* ≤ 0.10.

## 3. Results

### 3.1. Feed and Energy Intake during Gestation and Lactation

**Figure 2:**
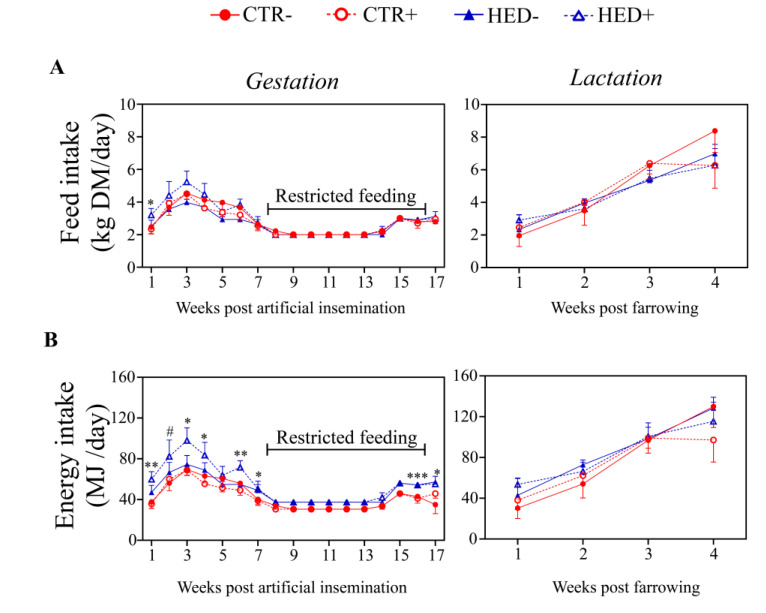
Effect of high-energy diet and spirulina supplementation on feed and energy intake of primiparous sows during gestation and lactation. (**A**) Feed intake during gestation and lactation (CTR- *n* = 5, CTR+ *n* = 6, HED- *n* = 5, and HED+ *n* = 4); (**B**) gross energy intake during gestation and lactation (CTR- *n* = 4, CTR+ *n* = 6, HED- *n* = 5, and HED+ *n* = 4). CTR: control diet; HED: high-energy diet. Data are presented in line graphs, where each point represents mean ± standard error of the mean (SEM). Significant differences were observed for dietary treatment (HED vs. CTR) and represented as * *p* < 0.05, ** *p* < 0.01, *** *p* < 0.001, and # *p* < 0.1 (trend). During the restricted feeding period, no statistical significance in energy intake was detected due to the absence of variance within groups.

In the first week post AI, feed intake was higher in the HED sows compared to the CTR sows (*p* = 0.047) (Figure 2A). For the rest of the gestation period and during lactation, the dietary energy density did not significantly affect the average daily dietary intake. The gross energy intake was higher in the HED sows compared to the CTR sows for almost the entire ad libitum period during gestation (*p* < 0.05), except in week five (Figure 2B). This resulted in an overall higher daily energy intake in the HED animals compared to the CTR animals (52.5 vs. 42.4 MJ/day, *p* < 0.001) during the gestation period. Similarly, the daily energy intake during lactation did not significantly differ between the experimental groups when considering weekly intervals, whereas regarding the entire lactation period, a higher daily energy intake was observed in the HED sows compared to the CTR sows (91.3 vs. 79.9 MJ/day, *p* = 0.033). The supplementation of Sp did not affect the feed and energy intake during both gestation and lactation.

### 3.2. Reproductive and Lactational Performance

Dietary energy density and Sp supplementation did not influence the gestation length, total litter size, total litter weight, or average piglet birth weight (Table 2). Numerically, the HED sows gave birth to one piglet less on average and showed a trend for one piglet less weaned (*p* = 0.071) than the CTR sows. A trend was also observed for a diet × Sp supplementation interaction on the piglets’ sex ratio (*p* = 0.072), with the HED+ sows (more male offspring) showing an increased male-to-female ratio compared to the HED- sows (more female offspring), while a similar male-to-female ratio with more male offspring was observed in the CTR- and CTR+ sows. Mortality at birth and until weaning after about 4 weeks was not significantly affected by either the HED or Sp supplementation. The total and daily milk yield did not differ between the four experimental groups.

### 3.3. Composition of the Colostrum

The gross composition of the colostrum (fat, protein, and lactose) as well as its gross energy content was not affected by either the dietary energy density or by Sp supplementation (Table 3). The same was true for the concentrations of the immunoglobulins IgA, IgG, and IgM. The FA profile of the colostrum was influenced by both the dietary energy density and Sp supplementation, but without exhibiting significant interactions. The colostrum lipids of the HED sows compared to the CTR sows contained a higher proportion of total and individual saturated FAs (SFA) including C15:0 (*p* = 0.017), C18:0 (*p* = 0.002), and C20:0 (*p* = 0.012). The HED led to lower colostrum proportions of linoleic acid (LA) (*p* = 0.029) and C20:2 *n*-6 (*p* < 0.001) compared to the CTR. The ratio of the polyunsaturated FAs (PUFA)/SFA tended to be lower (*p* = 0.077), while the ratio of the unsaturated FAs (UFA)/SFA was lower in the colostrum of the HED sows compared to that of the CTR sows (*p* = 0.022). The *n*-6/*n*-3 FA ratio was lower in the HED colostrum compared to the CTR colostrum (*p* = 0.001). The Sp supplementation resulted in higher proportions of C18:1 *cis*–11 (*p* = 0.030), C18:1 *cis*–13 (*p* = 0.030), and γ-linolenic acid (GLA) (*p* = 0.024) as well as a lower proportion of α-linolenic acid (ALA) (*p* = 0.048) in the colostrum compared to the colostrum from the non-supplemented sows.

## 4. Discussion

### 4.1. Effects of a High-Energy Diet

Feeding a high-energy or high-fat diet to gestating sows might affect their reproductive performance. The HED sows consumed more gross energy on average than the CTR sows during both the gestation and the lactation period [25]. The gestational length, number of piglets born, and number of piglets born alive remained unchanged in the HED group compared to the CTR group. Previous reports, however, showed that a high energy intake during gestation and lactation could result in larger litters with lower piglet birth weight [26,27]. In addition, rodent models indicated that high-fat diets can retard embryo development and cause embryonic toxicity and abortion [28,29]. These contrasting findings indicate that, in terms of the maternal excess energy intake, both the time point during gestation as well as the extent are relevant. A differing sensitivity of the offspring to environmental stimuli during in utero development has previously been reported [30,31]. Since in the present study we did not observe significant adverse effects of the HED on birth weight and litter size, and the extent of the increased energy intake was less pronounced during the restricted feeding period from gestational weeks 8 to 15, it can be assumed that the adverse effects of the maternal high energy intake might be particularly mediated during this respective period.

In addition to the high fat proportion, the HED also contained refined sugars and cholesterol which have both shown effects on reproductive parameters in previous studies. Dietary refined sugars can affect ovulation and ovarian functions [32] and dietary cholesterol may induce the activation of the liver X receptor in the ovaries, endometrium, and in the placenta, thus affecting follicle maturation, steroidogenesis, and cholesterol transport from the mother to the fetus [33,34,35,36]. To the best of the authors’ knowledge, no literature on the potential effects of dietary refined sugars and cholesterol on sow reproductive performance is available to date. Therefore, and because of the combined supply of fat, refined sugars, and cholesterol in the present study, no conclusions can be drawn on the potential impact of dietary refined sugars and cholesterol on HED sows and their offspring.

Oxidative stress has been reported to be associated with embryonic mortality [28,29], but at least at AI [25], no differences were observed in the antioxidative capacity of the sows from the present study. This apparent absence of increased oxidative stress in the HED sows compared to the CTR sows could contribute to explaining the absence of differences in litter size. The one less piglet weaned in the HED group compared to the CTR group could have been due to stillbirths and the pre-weaning mortality rates which, even though not significant, were on average almost 50% higher in the HED sows compared to the CTR sows. In the peripartum period, a previous study showed that sows fed a high-fat diet experienced changes in physiology and metabolism that caused elevated adiposity and systemic oxidative stress [37]. This phenomenon has been linked to increased neonatal piglet mortality [38]. Body weight [39] and back-fat thickness [25] did not differ between the HED sows and the CTR sows, while no accurate information about visceral adiposity is available for the sows of the present study. Hence, it cannot be excluded with certainty that the elevated fat accumulation around the birth canal might have been involved in the observed numerical increase in stillbirth mortality. In fact, such an accumulation of fat around the birth canal decreases its diameter, thus creating a physical obstacle that complicates farrowing and increases death cases in the birth process [40]. Additionally, Wang et al. [41] showed that an increased oxidative stress status has been observed in sows with a high stillbirth rate. Since the excess intake of fat and sugars has been demonstrated to increase oxidative stress [42], the increased energy intake of the HED animals in the present study might thus have increased oxidative stress, thereby slightly increasing offspring mortality. Still, these effects remain to be further investigated.

A high maternal intake of fat during gestation may also influence litter weight at birth. However, in the present study, no differences were observed in total litter size and birth weight. Contrary to that, Quiniou et al. [43] and Wang et al. [37] showed that the maternal intake of fat-rich diets during gestation can increase litter size and total litter weight compared to a low-fat group of sows. In contrast to Quiniou et al. [43] and Wang et al. [37] who used a soybean meal rich in PUFAs as the dietary fat source, the present study used hydrogenated palm oil rich in SFAs for the HED. The contrasting results between the present study and the studies performed by Quiniou et al. [43] and Wang et al. [37] suggest that the type of lipid sources and the degree of the dietary FA saturation in the maternal diet are decisive for the effects on litter size and offspring birth weight. Moreover, by increasing the maternal serum concentrations of triglycerides and reducing those of adiponectin, the intake of a high-fat diet during gestation may cause an up-regulation of placental nutrient transporters and subsequent fetal overgrowth. Such an association has not yet been shown in pigs but has been shown in rodent models [44]. Serum triglyceride concentrations did not differ in the gestating HED sows compared to the CTR sows of the present study [39], potentially because of the assumed high metabolic flexibility of these animals to effectively oxidize excess lipids [36]. The absence of effects in the sows’ lipid profiles, at least at the investigated time points, might be one of the reasons why no fetal overgrowth and thus consequently also no effect on the gestation length was observed in the present pig model.

Excessive energy intake during late gestation may decrease milk production in the case of a feed intake depression after farrowing [45]. However, in the present study, despite the overall higher energy intake during gestation, the feed intake during lactation, the calculated total milk production, the daily milk yield per piglet, and the analysed colostrum gross composition were not significantly affected in the present study by the HED. An excessive energy intake may also modify milk composition during the lactation period [45]. This is especially the case when fat of a distinct composition is used for increasing the energy density of the diet, as was performed in the present study, and this may also influence the composition of the colostrum. We used hydrogenated palm oil as a major fat source in the HED. Hydrogenated palm oil is particularly rich in SFAs, especially C18:0 and C16:0 [46], in line with the analysed FA composition of our HED. Despite the high desaturase activity observed in the mammary gland of sows [47,48], the HED intake accordingly resulted in higher SFA proportions of the colostrum lipids compared to the CTR. This was especially obvious in the proportion of C18:0, which was increased from the CTR to the HED by a factor of 13. The large differences in the dietary C16:0 proportions were, however, not observed in the colostrum.

### 4.2. Effects of Spirulina Supplementation

In the present study, Sp did not influence reproductive performance. Similar to the HED, Sp did not influence milk yield or the gross composition of the colostrum. These results contrast with the observations made by Shimkus et al. [49], who reported an increased birth weight, milk yield, as well as increased fat, protein, and lactose concentrations in the milk from sows, and a decreased pre-weaning mortality in the litter from sows that were supplemented with spirulina, but with a much lower dose (only 2 g spirulina per day) than was used in the present study. However, this does not allow one to conclude that lower spirulina doses result in more pronounced effects, since other relevant information about the diet, breed of the animals, and rearing conditions is not available in Shimkus et al. [49]. Further, the Sp effects on the colostrum FA profile were overall less pronounced than those of the dietary energy density. There are two characteristic Sp FAs, namely LA and GLA. Different from the proportion of the GLA, which was higher in the colostrum of the Sp supplemented versus non-supplemented sows, there was no response to the intake of lipids with a higher LA proportion. Since the present manuscript is the first to report FA profiles in sow colostrum following Sp intake, the results cannot be compared with the literature data obtained from pigs. However, a similar increase in the GLA proportion of milk fat with Sp supplementation was found in dairy cows [50]. It can be assumed that the presence of GLA in the colostrum of the Sp supplemented sows might have effects on the suckling offspring due to its importance in inflammatory and lipid metabolism pathways [51]. Therefore, the transfer of GLA from sow to offspring via milk may be beneficial for the piglets, a hypothesis which should be further investigated in detail in the future.

### 4.3. Interaction of Maternal Spirulina Supplementation and Maternal diet

Spirulina is known to exert antioxidant effects and may thus have the potential to attenuate the effects of a HED and improve the reproductive performance of sows. In the present study, this was not the case, possibly because of the absence of significant adverse effects in the HED sows. A trend was observed for an interaction of dietary energy density and Sp supplementation, suggesting that Sp supplementation may have influenced the offspring sex ratio in a diet-specific manner. Accordingly, maternal nutrition has previously been demonstrated to skew the offspring sex ratio in cows, sheep, and rodents (as reviewed by Naidu et al. [52]). Nutritional and thus non-invasive strategies to pre-select or at least increase the likeliness of the desired offspring sex may further optimize livestock production and help meet the increasing demand for animal-derived foods for human nutrition [53]. In the present study, the HED- sows gave birth to more female than male offspring, whereas all other groups had more male than female offspring, showing for the first time that Sp may have skewed the sex ratio in the HED group to be more similar to that of the CTR sows. Contrary to the comparably higher number of female piglets in our HED- group, Alexenko et al. [54] reported for mice that an SFA-rich diet skewed the sex ratio toward more males. Rosenfeld et al. [55] reported that the sex ratio of mouse pups born to mothers fed a diet very high in SFAs was similarly low, with more females than males in the litters. Apart from the proportions of the SFAs and UFAs, the sex ratio of the offspring may also be affected by the type of UFA, where *n*-6 PUFA may play a specific role. Literature on the effects of dietary *n*-6 PUFA on the offspring’s sex ratio is contradictory in different species. Feeding gestating dams with diets rich in *n*-6 PUFA has resulted in a skewing of the sex ratio toward more females than males in rodents and sheep [56,57,58]. However, a shift toward more males was observed in cows fed a diet rich in *n*-6 LA [59]. Spirulina is particularly rich in *n*-6 PUFA due to its high prevalence of LA and GLA. Considering the low amount of Sp supplemented to our sows that, in addition, was started only 3 days before AI, a causative association of the Sp *n*-6 PUFA and the observed numerical increase in male compared to female offspring in the HED sows would be rather remarkable. Future studies with larger group sizes should be performed to confirm the present findings and investigate the underlying mechanisms for the potential effect of Sp on the offspring sex ratio in HED-fed pigs.

Since milk represents the first dietary source of newborn offspring, changes in the milk composition may not only affect the offspring’s growth during the suckling period but may also have effects that persist even later in life. This phenomenon is also referred to as “fetal programming” [60]. Indeed, the offspring of the sows from the present study showed a response to the Sp supplementation of their dams later in growth; however, this was in the direction of a lower growth performance [17].

## 5. Conclusions

In conclusion, feeding a diet particularly rich in fat, sugars, and cholesterol to pigs before and during gestation as well as during lactation did not significantly affect their reproductive and lactational performance. In fact, despite the overall higher average gross energy intake during gestation and lactation in the HED sows compared to the CTR sows, the extent of the increased energy intake was limited during an eight-week period starting around mid-gestation due to a restricted feeding regime. This might have contributed to the few significant effects observed following the HED intake. However, different metabolic responses that may have contributed to the absence of effects still remain to be investigated. Our hypothesis (i) had to be rejected by the present study results. Still, the trend for a lower vitality of piglets when sows were fed such a diet, as evidenced by the tendency for a lower number of weaned piglets, has to be carefully monitored. Hypothesis (ii) was confirmed since feeding a HED during gestation and lactation modified the FA composition of the colostrum composition, though not its gross nutrient composition and immunoglobulin concentrations. The changes in the FA composition may have the potential to impact offspring metabolic health and future performance. Lacking the effects of dietary energy density, Sp supplementation was not able counteract any detrimental effects. Hypothesis (iii) was still partially confirmed because the sex ratio tended to be regulated by Sp supplementation in a diet-dependent manner. The few effects of Sp supplementation on colostrum FA composition occurred independently of the energy density of the diet. Overall, the present results suggest that a diet with a particularly high energy density, with hydrogenated palm oil and refined sugars as the major dietary fat and carbohydrate sources, can be used in the nutrition of highly prolific sows without larger risks of adverse effects. Further investigations with larger animal numbers have to confirm whether higher dosages of Sp may favorably influence reproductive and lactational performance. It also seems worth investigating whether there are also differences in the FA composition in ripe milk as induced by both the HED and Sp supplementation and what effects these changes may have on the physiology of the growing offspring.

## Figures and Tables

**Table 1:** Ingredients and composition of the experimental diets.

	Gestation Diet		Lactation Diet		
	CTR	HED	CTR_L_	HED_L_	Spirulina
*Ingredients (g/kg as fed)*					
Hydrogenated palm oil	-	150.00	-	150.00	-
Saccharose	-	200.00	-	200.00	-
d-Fructose	-	150.00	-	150.00	-
Cholesterol	-	2.00	-	2.00	-
Corn germ	590.00	298.00	488.00	200.00	-
Wheat	300.00	-	300.00	-	-
Soybean meal	45.00	130.00	145.00	217.00	-
Lignocellulose	26.60	29.00	26.60	29.00	-
Monocalcium phosphate	13.00	20.00	11.20	20.00	-
Calcium carbonate	11.00	6.00	11.60	6.00	-
NaCl	6.20	6.00	6.20	6.00	-
Vitamin and mineral premix †	5.00	5.00	5.00	5.00	-
l-Lysine	2.30	2.50	3.60	5.40	-
dl-Methionine	-	1.00	0.70	1.90	-
dl-Tryptophan	0.20	0.40	0.50	1.00	-
l-Threonine	0.70	1.30	1.80	3.40	-
Valine	-	-	-	2.20	-
*Chemical composition (g/kg dry matter if not stated otherwise)*					
Total ash	47.80	47.14	54.94	54.32	90.20
Crude protein	110.00	93.52	150.95	162.33	638.00
Ether extract	26.83	158.17	29.14	143.40	47.06
Starch	462.00	93.00	408.00	74.50	51.00
Total sugars	46.50	359.00	51.50	326.00	<0.5
Crude fiber	6.71	8.67	6.17	8.07	n.a.
Cholesterol	0.03	0.48	0.02	0.45	*n*.a
Gross energy (MJ/kg dry matter)	13.41	17.17	12.96	16.75	n.a.
*Fatty acid composition (g/100 g total FA)* ^§^					
C12:0	0.14	0.64	0.30	0.08	0.03
C14:0	0.15	0.91	0.12	0.11	0.17
C16:0	15.96	30.69	12.32	5.92	42.97
*iso* C16:0	0.06	0.00	0.06	0.00	1.93
C16:1 *n*-7	0.15	0.01	0.12	0.03	5.44
C16:1 *x*	0.34	0.00	0.04	0.00	0.05
C17:1	0.03	0.00	0.04	0.03	0.33
C18:1 *cis*-9	26.04	3.31	21.82	2.86	1.62
C18:1 *cis*-11	0.88	0.11	0.67	0.12	0.73
C18:2 *n*-6 (LA)	47.60	5.04	45.25	5.07	16.58
C18:3 *n*-3 (ALA)	1.93	0.34	2.28	0.68	0.43
C18:3 *n*-6 (GLA)	0.00	0.00	0.00	0.00	23.23
C20:0	0.49	0.89	0.64	2.00	0.09
C20:1 *n*-9	0.42	0.04	0.34	0.06	0.12
C20:2 *n*-6	0.11	0.01	0.05	0.00	0.30
C20:3 *n*-6	0.00	0.00	0.00	0.00	0.38
C22:0	0.22	0.35	0.35	1.15	0.00
∑ Saturated FA	22.02	90.34	29.11	91.11	49.22
∑ Monounsaturated FA	28.02	4.26	23.3	3.14	8.85
∑ Polyunsaturated FA	49.96	5.40	47.62	5.75	41.93
∑ *n*-6 FA	47.71	5.05	45.30	5.07	40.40
∑ *n*-3 FA	1.90	0.34	2.27	0.68	0.45
*n*-6/*n*-3 FA ratio	25.13	14.74	19.68	7.44	73.10

CTR: control diet; FA: fatty acids; HED: high-energy diet; n.a.: not analysed. ^†^ For details on the composition of the premix see [17]. ^§^ Only fatty acids (FA) with a proportion of >0.3 g/100 g total FA in at least one of the feed items are displayed.

**Table 2:** Effect of a high-energy diet and spirulina supplementation on reproductive and lactational performance of the sows.

Diet (D)	CTR		HED		SEM	*Significance*		
Spirulina (Sp)	-	+	-	+		D	Sp	D × Sp
Gestation length (d) ^1^	116.20	115.80	116.20	114.25	1.437	*n.s.*	*n.s.*	*n.s.*
*Litter size (piglets)* ^1^								
Total ^†^	13.20	12.40	11.66	12.60	2.070	*n.s.*	*n.s.*	*n.s.*
Born alive	11.20	11.82	10.12	11.15	2.498	*n.s.*	*n.s.*	*n.s.*
Weaned	10.15	10.35	9.00	9.25	2.442	*#*	*n.s.*	*n.s.*
Sex ratio (male:female) ^1^	1.50	1.12	0.83	1.77	0.453	*n.s.*	*n.s.*	*#*
Total litter weight (kg) ^1,^^‡^	20.62	19.02	19.44	18.55	3.233	*n.s.*	*n.s.*	*n.s.*
Average birth weight (kg) ^1,^^‡^	1.670	1.580	1.740	1.560	0.130	*n.s.*	*n.s.*	*n.s.*
*Mortality (%)* ^1^								
Foetal deaths	12.31	4.11	13.48	16.36	6.159	*n.s.*	*n.s.*	*n.s.*
Until weaning	8.58	9.43	10.63	19.06	6.331	*n.s.*	*n.s.*	*n.s.*
*Milk yield (kg)* ^2^								
Total	325.22	283.38	280.88	292.35	65.834	*n.s.*	*n.s.*	*n.s.*
Per piglet per day	1.07	0.75	1.01	0.98	0.159	*n.s.*	*n.s.*	*n.s.*

CTR: control diet; HED: high-energy diet; *n*.s.: not significant. ^1^ CTR- *n* = 5, CTR+ *n* = 6, HED- *n* = 5, and HED+ *n* = 4. ^2^ CTR- *n* = 4, CTR+ *n* = 6, HED- *n* = 5, and HED+ *n* = 4. ^†^ Includes piglets born alive, stillborn, and deformed piglets. ^‡^ Includes piglets born alive and stillborn. Data are presented as least square means ± standard error of the mean (SEM). Trends are defined as # 0.05 > *p* < 0.1.

**Table 3:** Effect of a high-energy diet and spirulina supplementation on colostrum composition of the sows.

Diet (D)	CTR		HED		SEM	*Significance*		
Spirulina (Sp)	-	+	-	+		D	Sp	D × Sp
Fat (g/100 g)	9.77	9.07	8.26	6.21	1.870	*n.s.*	*n.s.*	*n.s.*
Protein (g/100 g)	15.92	16.37	15.81	15.29	0.751	*n.s.*	*n.s.*	*n.s.*
Lactose (g/100 g)	0.88	1.07	1.05	1.19	0.148	*n.s.*	*n.s.*	*n.s.*
Estimated energy content (MJ/kg)	8.36	8.21	7.77	6.87	0.873	*n.s.*	*n.s.*	*n.s.*
IgA (mg/mL)	6.92	7.53	6.37	7.31	1.330	*n.s.*	*n.s.*	*n.s.*
IgG (mg/mL)	57.61	56.03	60.91	58.26	8.462	*n.s.*	*n.s.*	*n.s.*
IgM (mg/mL)	2.68	3.11	2.29	2.49	0.511	*n.s.*	*n.s.*	*n.s.*
*Fatty acid (FA) composition (g/100 g total FA)* ^†^								
C14:0	1.61	1.41	1.68	1.47	0.329	*n.s.*	*#*	*n.s.*
C15:0	0.09	0.07	0.14	0.11	0.038	*	*n.s.*	*n.s.*
C16:0	25.17	25.44	25.00	25.53	1.542	*n.s.*	*n.s.*	*n.s.*
*iso* C16:0	0.89	0.93	0.88	0.94	0.514	*n.s.*	*n.s.*	*n.s.*
C16:1 *n*-7	2.91	2.73	2.85	3.43	0.465	*n.s.*	*n.s.*	*n.s.*
C17:0	0.21	0.19	0.26	0.20	0.064	*n.s.*	*#*	*n.s.*
C17:1	0.17	0.17	0.21	0.19	0.048	*#*	*n.s.*	*n.s.*
C18:0	10.02	10.61	12.86	12.33	3.610	**	*n.s.*	*n.s.*
C18:1 *cis*-9	36.97	38.76	37.33	37.99	1.682	*n.s.*	*n.s.*	*n.s.*
C18:1 *cis*-11	3.06	3.24	2.63	3.19	0.331	*#*	*	*n.s.*
C18:1 *cis*-13	0.12	0.15	0.12	0.15	0.019	*n.s.*	*	*n.s.*
C18:2 *n*-6 (LA)	14.28	12.39	11.31	9.59	2.013	*	*n.s.*	*n.s.*
C18:3 *n*-6 (GLA)	0.12	0.19	0.11	0.14	0.094	*n.s.*	*	*n.s.*
C18:3 *n*-3 (ALA)	0.65	0.48	0.59	0.53	0.164	*n.s.*	*	*n.s.*
C20:0	0.13	0.15	0.20	0.17	0.024	*	*n.s.*	*#*
C20:1 *cis*-9	0.53	0.53	0.44	0.48	0.232	*#*	*n.s.*	*n.s.*
C20:2 *n*-6	0.33	0.31	0.20	0.19	0.051	***	*n.s.*	*n.s.*
C20:3 *n*-6	0.18	0.17	0.16	0.19	0.033	*n.s.*	*n.s.*	*n.s.*
C20:4 *n*-6	1.26	1.27	1.87	1.58	0.795	*n.s.*	*n.s.*	*n.s.*
C20:5 *n*-3	0.13	0.11	0.15	0.15	0.090	*n.s.*	*n.s.*	*n.s.*
C22:5 *n*-3	0.17	0.16	0.20	0.19	0.046	*n.s.*	*n.s.*	*n.s.*
∑SFA	38.63	39.15	40.90	41.14	1.596	*	*n.s.*	*n.s.*
∑MUFA	44.05	45.94	44.14	45.76	1.899	*n.s.*	*n.s.*	*n.s.*
∑PUFA	17.21	15.29	15.15	12.89	1.992	*n.s.*	*n.s.*	*n.s.*
MUFA/SFA ratio	1.14	1.18	1.08	1.13	0.065	*n.s.*	*n.s.*	*n.s.*
PUFA/SFA ratio	0.45	0.40	0.38	0.31	0.059	*#*	*n.s.*	*n.s.*
UFA/SFA ratio	1.61	1.57	1.45	1.45	0.094	*	*n.s.*	*n.s.*
∑ *n*-3 FA	1.02	0.84	1.10	0.96	0.114	*n.s.*	*n.s.*	*n.s.*
∑ *n*-6 FA	16.01	14.29	13.87	11.63	1.908	*n.s.*	*n.s.*	*n.s.*
*n*-6/*n*-3 FA ratio	15.62	17.00	12.42	12.60	1.372	**	*n.s.*	*n.s.*

ALA: α-linolenic acid; CTR: control diet; GLA: γ-linolenic acid; HED: high-energy diet; IG: immunoglobulin; LA: linoleic acid; MUFA: monounsaturated fatty acids; *n*.s.: not significant; PUFA: polyunsaturated fatty acids; SFA: saturated fatty acids; UFA: unsaturated fatty acids. Data are presented as least square means ± standard error of the mean (SEM). Statistical significances were set at * *p* < 0.05, ** *p* < 0.01, and *** *p* < 0.001. Trends were defined as # 0.05 > *p* < 0.10. For all parameters analysed: CTR- *n* = 4, CTR+ *n* = 6, HED- *n* = 5, and HED+ *n* = 4. ^†^ Only fatty acids (FA) with a proportion of >0.1 g/100 g total FA in at least one of the groups or tissues are displayed.

## Data Availability

The datasets used and analysed during the current study are available from the corresponding author on reasonable request.
